# Serum Copper-to-Zinc Ratio and Oxidative Stress Are Associated with Anemia in Older Adults with Cardiovascular–Kidney–Metabolic Syndrome

**DOI:** 10.3390/ijms27135840

**Published:** 2026-06-28

**Authors:** Giuseppe Bruschetta, Guido Gembillo, Lorenzo Lo Cicero, Angela D’Ascola, Fabio Bruno, Andrea Corsonello, Domenico Santoro, Mirko Di Rosa, Luca Soraci

**Affiliations:** 1 Biochemistry Unit, Department of Veterinary Sciences, University of Messina, Viale Palatucci, 13, 98168 Messina, Italy; fabio.bruno@unime.it; 2 Unit of Nephrology and Dialysis, Department of Clinical and Experimental Medicine, University of Messina, 90125 Messina, Italy; guidogembillo@live.it (G.G.); lorenzolocicero95@gmail.com (L.L.C.); dsantoro@unime.it (D.S.); 3 Department of Clinical and Experimental Medicine, University of Messina, Policlinico Universitario, Via Consolare Valeria 1, 98125 Messina, Italy; adascola@unime.it; 4 Unit of Geriatric Medicine and Clinical Epidemiology, INRCA IRCCS, Cda Muoio Piccolo, 87100 Cosenza, Italy; a.corsonello@inrca.it (A.C.); m.dirosa@inrca.it (M.D.R.); l.soraci@inrca.it (L.S.); 5 Department of Pharmacy, Health and Nutritional Sciences, University of Calabria, Arcavacata di Rende, 87036 Cosenza, Italy

**Keywords:** CKM syndrome, oxidative stress, Cu/Zn ratio, selenium, SOD1, RDW, uric acid, anemia, NHANES, pro-oxidant

## Abstract

Chronic oxidative stress is a molecular hallmark of cardiovascular–kidney–metabolic (CKM) syndrome, yet its contribution to CKM-associated anemia beyond erythropoietin deficiency and iron restriction is poorly characterized. The serum copper-to-zinc (Cu/Zn) ratio reflects impaired Cu/Zn-SOD1 antioxidant capacity and inflammatory trace-element imbalance, but its relationships with circulating redox biomarkers and its hematological relevance in CKM syndrome has never been explored in a community-dwelling cohort of older adults. We analyzed 2391 NHANES 2011–2016 participants ≥ 50 years of age with CKM stage I-IV. To explore whether the serum Cu/Zn ratio was associated with oxidative stress and immunomodulatory biomarkers as well as with the odds of anemia, we used survey-weighted Spearman correlations, linear regression (outcome: hemoglobin), and logistic regression (outcome: anemia); multivariate models were adjusted for a panel of antioxidant or immunomodulatory biomarkers (selenium, vitamin D), pro-oxidant biomarkers (lead, cadmium, cotinine, uric acid), red cell distribution width (RDW) as a composite biomarker of erythrocyte stress, neutrophil-to-lymphocyte ratio (NLR), CKM stage, and comorbidities. The molecular targets of the nine biomarkers were mapped onto a protein–protein interaction network using the STRING database v12.0 to contextualize regression findings within a systems biology framework. Anemia was present in 205 participants (8.6%). The Cu/Zn ratio was inversely correlated with the antioxidant marker selenium (r = −0.19; *p* < 0.001) and positively correlated with the pro-oxidant markers RDW (r = +0.21; *p* < 0.001) and cadmium (r = +0.10; *p* < 0.001), consistent with its role as a hub within the CKM redox network. In fully adjusted models, a higher Cu/Zn ratio was independently associated with prevalent anemia (OR = 2.94; 95% CI: 1.61–5.37) and lower hemoglobin (β = −0.55 g/dL); among included biomarkers, selenium and cadmium were independently protective (OR = 0.76 per 10 µg/L and 0.23 per µg/dL, respectively), and RDW and uric acid were independently harmful (OR = 2.20 per 1% and 1.33 per mg/dL, respectively). The Cu/Zn ratio correlated with both antioxidant depletion and pro-oxidant accumulation in CKM syndrome and was independently associated with anemia within this oxidative network. Together with selenium, cadmium, RDW, and uric acid, it defines an oxidative stress-driven hematological pathway that may contribute to the development and progression of anemia in patients with CKM syndrome.

## 1. Introduction

Cardiovascular–kidney–metabolic (CKM) syndrome represents a systemic disorder characterized by the progressive and chronic deterioration of kidney, cardiovascular, and metabolic functions, placing affected individuals at a substantial risk of poor prognosis [1,2]. The 2023 American Heart Association Presidential Advisory defined the main characteristics of the syndrome and provided a classification into four progressive stages [1]: from stage 0, characterized by the absence of disease and metabolic risk factors, through stage 1–2 with substantial metabolic risk, to stages 3-4, characterized by subclinical and overt CKM syndrome [3,4]. Given the involvement of multiple organs, multidisciplinary interventions are deemed necessary to delay the progression of the disease [1,2].

The importance of CKM in older populations is substantial: between 2011 and 2020, nearly 90% of community-dwelling adults in the United States met criteria for CKM syndrome, and about 15% were affected by advanced disease [3,4]; individuals at stage 4 face a fourfold increased risk of all-cause mortality and lose more than 15 life-years compared with those at stage 0 [5].

Despite their distinct disease trajectories, all CKM stages share a common molecular hallmark: chronic oxidative stress [6,7], driven by sustained superoxide flux from multiple interconnected pathways, including Nuclear Factor kappa-light-chain-enhancer of activated B cells (NF-κB) activation [8], renin–angiotensin–aldosterone system (RAAS) hyperactivation [9], accumulation of advanced glycation end-products (AGEs) with subsequent engagement of their receptor RAGE [10,11,12], and mitochondrial dysfunction [13].

One of the most clinically significant consequences of the chronic oxidative stress in CKM is the development of anemia, whose prevalence and severity both increase with advancing CKM stage [14,15,16]. Its pathogenesis involves multiple canonical pathways: accumulation of uremic toxins and mitochondrial dysfunction in peritubular cells suppress the synthesis of erythropoietin (EPO) via HIF-2α–dependent pathways [17]; IL-6-mediated induction of hepcidin drives subsequent degradation of ferroportin (SLC40A1) and iron-restricted erythropoiesis, even with normal iron stores [18,19]; finally, reactive oxygen species (ROS) may directly damage erythrocyte membranes [20].

However, the persistence of anemia despite adequate correction of these canonical mechanisms suggests that additional measurable contributors remain to be characterized. Among such contributors, the serum copper-to-zinc ratio (Cu/Zn) and the imbalance between other pro-oxidant and antioxidant compounds may be critical.

The serum Cu/Zn is a composite measure of inflammation and antioxidant capacity [21,22]. Copper and zinc are obligate catalytic cofactors of the Cu/Zn superoxide dismutase (SOD1), the most abundant cytosolic antioxidant enzyme in erythrocytes and in all nucleated cells [23,24]. The two elements have different roles: depletion of copper blocks the catalytic cycling entirely, whereas depletion of zinc impairs the stability of SOD1 homodimer independently of copper [23]. Decreased superoxide dismutation promotes the reaction of superoxide with nitric oxide (NO), thus generating peroxynitrite (ONOO^−^); this compound initiates the lipid peroxidation of the spectrin–ankyrin membrane and the phosphatidylserine externalization, which may shorten erythrocyte lifespan and impair their deformability [25]. Furthermore, copper excess may instead influence the activity of ceruloplasmin and hephaestin ferroxidase, both required for the Fe^2+^ to Fe^3+^ oxidation and subsequent transferrin loading; excess copper also triggers cuproptosis in fibrotic renal tubular cells overexpressing copper transporter 1 (CTR1) (SLC31A1) and activates metal-regulatory transcription factor 1 (MTF1)-driven hepcidin upregulation, creating a feedforward loop with the IL-6–ferroportin axis.

Previous studies have highlighted that the Cu/Zn ratio independently predicts incidence of chronic kidney disease (CKD) in type 2 diabetes [26,27]. Furthermore, higher Cu/Zn values were associated with lower hemoglobin and prevalent anemia in patients with CKD undergoing maintenance hemodialysis [28]. However, the association between Cu/Zn and anemia in patients with CKM syndrome has not been tested, leaving a gap in current evidence. Moreover, prior studies evaluated the Cu/Zn ratio in isolation, without simultaneously adjusting for multiple pro-oxidants and antioxidant confounders that may shape this risk. Among antioxidants, selenium, the catalytic cofactor of glutathione peroxidase (GPx) is very relevant, as it operates enzymatically downstream of SOD1 in the same ROS-scavenging cascade; its depletion in CKD has been previously linked to increased erythrocyte fragility and anemia [29]. Conversely, pro-oxidant exposures such as cadmium, lead, and cotinine may damage erythrocyte membranes through mechanisms independent of the Cu/Zn axis [30,31,32]; finally, red cell distribution width (RDW) and uric acid may contribute to erythrocyte vulnerability in CKM [33,34].

The present study addresses these gaps by analyzing data from 2391 older community-dwelling adults with CKM stage I-IV, with two different aims: first, we tested whether the serum Cu/Zn ratio was independently associated with circulating concentrations of pro-oxidant biomarkers (lead, cadmium, cotinine, uric acid), antioxidant and/or immunomodulatory biomarkers (selenium, vitamin D), a composite erythrocyte stress marker represented by RDW, and neutrophil-to-lymphocyte ratio (NLR); second, we assessed whether the serum Cu/Zn ratio was associated with prevalent anemia and lower hemoglobin concentration, after simultaneous adjustment for such biomarkers, CKM stages, and chronic diseases.

## 2. Results

### 2.1. Baseline Characteristics

The final sample included 2391 participants with a mean (SD) age of 63.6 (9.3) years and a slight predominance of women (51%); 205 participants (8.6%) met World Health Organization (WHO) criteria for anemia. The baseline characteristics of the overall sample and of the sample stratified by anemia are reported in Table 1.

Patients with anemia were significantly older and had a higher prevalence of cardiovascular disease, hypertension, type 2 diabetes mellitus, and stroke, compared with non-anemic patients; they were also characterized by worse kidney function (lower eGFR and higher ACR), and a higher prevalence of advanced CKM stages (37.6% vs. 14.5%; *p* < 0.001). Among trace elements, serum copper was higher (126.3 vs. 116.8 µg/dL), zinc was lower (76.5 vs. 83.0 µg/dL), and the Cu/Zn ratio was higher (1.7 vs. 1.4; all *p* < 0.001) in patients with anemia compared with those without anemia; in addition, both selenium and cadmium were lower in anemic patients. Conversely, uric acid (6.0 vs. 5.5 mg/dL; *p* = 0.003) and RDW (14.8% vs. 13.4%; *p* < 0.001) were both higher in anemic patients compared with non-anemic participants. Vitamin D, lead, cotinine, and neutrophil-to-lymphocyte ratio (NLR) did not differ significantly between groups (Table 1).

**Figure 1:**
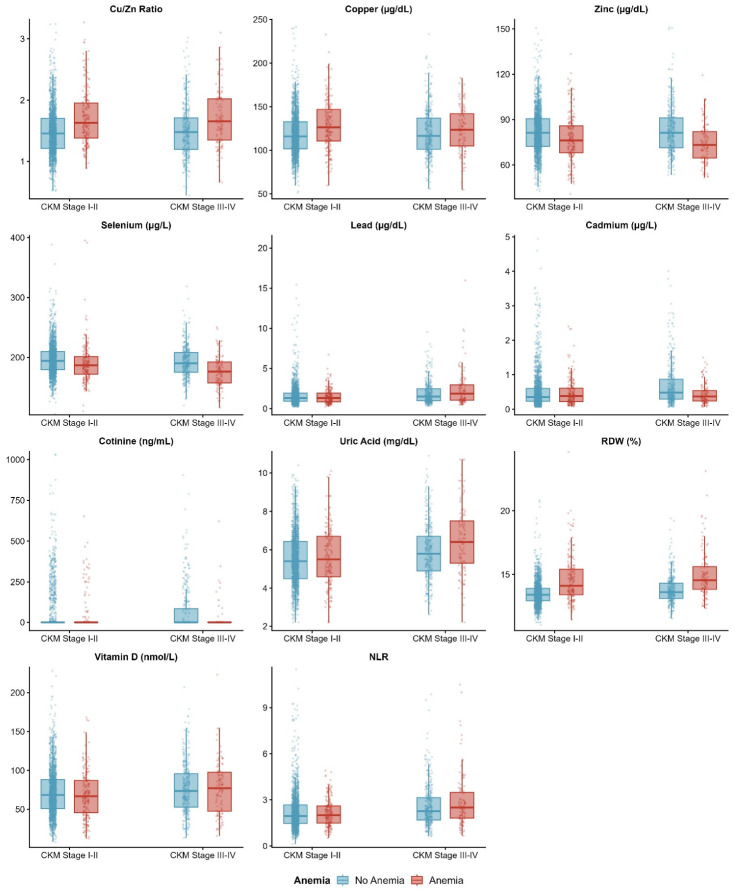
Distribution of circulating biomarkers by CKM syndrome stage and by anemia status. Boxplots show the median (horizontal line) and interquartile range (box). Each panel shows a biomarker, stratified by CKM syndrome stage (CKM Stage I–II vs. CKM Stage III–IV) on the *x*-axis and anemia status (blue: No Anemia; red: Anemia) as fill color. Individual participant values are overlaid as jittered dots.

Across CKM stages, participants with anemia showed consistently higher median (IQR) Cu/Zn ratios than those without anemia: from 1.68 (0.44) and 1.44 (0.36) in stage 1–2 to 1.71 (0.47) and 1.46 (0.40) in stage 3–4, respectively; they also had higher copper, higher uric acid, and higher RDW compared with non-anemic participants. Conversely, selenium and zinc were lower in anemic participants at both stages, with the selenium deficit widening at stage 3–4 (172.3 [25.4] µg/L in anemic participants vs. 193.5 [24.6] µg/L in non-anemic participants). The Cu/Zn–anemia gap was consistent across stages, while the uric acid difference was most pronounced at stage 3–4 (6.75 [1.75] vs. 5.74 [1.40] mg/dL), suggesting greater xanthine oxidoreductase (XOR)-driven oxidative burden in advanced CKM. Cadmium showed an inverse pattern, with higher levels in non-anemic participants at stage 3–4. Lead, cadmium, and vitamin D showed modest or inconsistent differences across groups. This was confirmed by the distribution of medians and IQRs of biomarker concentrations (Figure 1).

### 2.2. Cu/Zn Ratio and the Oxidative Stress Biomarker Panel

Survey-weighted Spearman correlations between biomarkers, the Cu/Zn ratio, and hemoglobin are reported in Table 2 and visualized in Figure S1.

Three distinct correlation patterns emerged. First, selenium showed the most coherent antioxidant signal: a significant inverse correlation with the Cu/Zn ratio (r = −0.20; *p* < 0.001) and the strongest positive correlation with hemoglobin (r = +0.25; *p* < 0.001) in the panel, consistent with joint depletion of the SOD1–GPx enzymatic axis. Second, RDW showed the strongest hematological association of all biomarkers: a significant positive correlation with the Cu/Zn ratio (r = +0.21; *p* < 0.001) and the strongest negative correlation with hemoglobin (r = −0.32; *p* < 0.001), supporting RDW as an integrative downstream marker of Cu/Zn-driven oxidative erythrocyte stress. Third, cadmium was positively correlated with the Cu/Zn ratio (r = +0.10; *p* < 0.001) and positively but weakly correlated with hemoglobin (r = +0.07; *p* < 0.001). Vitamin D showed a weak but significant inverse correlation with the Cu/Zn ratio (r = −0.07; *p* = 0.01) but no significant correlation with hemoglobin (r = −0.02; *p* = 0.397). Lead, cotinine, uric acid, and NLR were not significantly correlated with the Cu/Zn ratio (all *p* > 0.05), though uric acid (r = +0.09; *p* = 0.01) and cotinine (r = +0.15; *p* < 0.001) were weakly but significantly correlated with hemoglobin.

### 2.3. Cu/Zn Ratio, Oxidative Stress Biomarkers, and Hemoglobin Concentration

Survey-weighted linear regression confirmed independent associations between the Cu/Zn ratio and hemoglobin (Table 3).

In Model A (adjusted for age and sex), each unit increment in the Cu/Zn ratio corresponded to a 0.79 g/dL lower hemoglobin (95% CI: −0.98 to −0.60; *p* < 0.001); this estimate was partially attenuated in Model B (β = −0.55; 95% CI: −0.75 to −0.35; *p* < 0.001), which was additionally adjusted for obesity, cardiovascular disease, hypertension, diabetes, cancer, stroke, CKM stage III–IV, and all panel biomarkers. Among covariates in Model B, selenium was independently and positively associated with hemoglobin (β = +0.10 per 10 µg/L increment; 95% CI: 0.07–0.13; *p* < 0.001); RDW carried the largest absolute effect estimate of any biomarker (β = −0.31 per 1% increment; 95% CI: −0.39 to −0.23; *p* < 0.001), reflecting its role as an integrative downstream marker of impaired erythropoiesis. Cadmium showed a significant positive association with hemoglobin in both Model A (β = +0.35; *p* < 0.001) and Model B (β = +0.42; *p* < 0.001). After applying Benjamini–Hochberg false discovery rate (FDR) correction across the nine biomarkers in linear regression models, Cu/Zn ratio, selenium, RDW, and cadmium remained significantly associated with hemoglobin in models A and B (all FDR-adjusted *p*-values <0.001).

Lead, vitamin D, cotinine, and uric acid were not significantly associated with hemoglobin in the fully adjusted model. Tertile analysis of the Cu/Zn ratio confirmed a monotonic gradient: compared with T1 (lowest tertile), T2 corresponded to a β = −0.29 (95% CI: −0.49 to −0.08) and T3 to β = −0.27 (95% CI: −0.48 to −0.07) in Model B (Table S1).

### 2.4. Cu/Zn Ratio, Oxidative Stress Biomarkers, and Anemia Prevalence

Survey-weighted logistic regression confirmed independent associations between the Cu/Zn ratio and prevalent anemia (Table 4).

Each unit increment in the Cu/Zn ratio carried 2.88-fold odds of anemia in Model A (95% CI: 1.58–5.27) and 2.94-fold in the fully adjusted Model B (95% CI: 1.61–5.37). Selenium independently reduced anemia odds in both models (Model B: OR = 0.76 per 10 µg/L; 95% CI: 0.67–0.86; *p* < 0.001). RDW independently increased anemia odds (Model B: OR = 2.20 per 1% increase; 95% CI: 1.68–2.86; *p* < 0.001), similarly to uric acid (Model B: OR = 1.33 per mg/dL; 95% CI: 1.07–1.65; *p* = 0.009). Cadmium was independently and inversely associated with anemia (Model B: OR = 0.23 per µg/L; 95% CI: 0.09–0.56; *p* < 0.001). Lead, vitamin D, cotinine, and NLR were not independently associated with anemia in either model. Study results were confirmed after applying Benjamini–Hochberg FDR correction. Tertile analysis (Table S2) revealed that Cu/Zn T2 and T3 both carried significant odds in all models, without a strict monotonic pattern from Model A to B.

### 2.5. Protein–Protein Interaction Network of Biomolecular Targets

**Figure 2:**
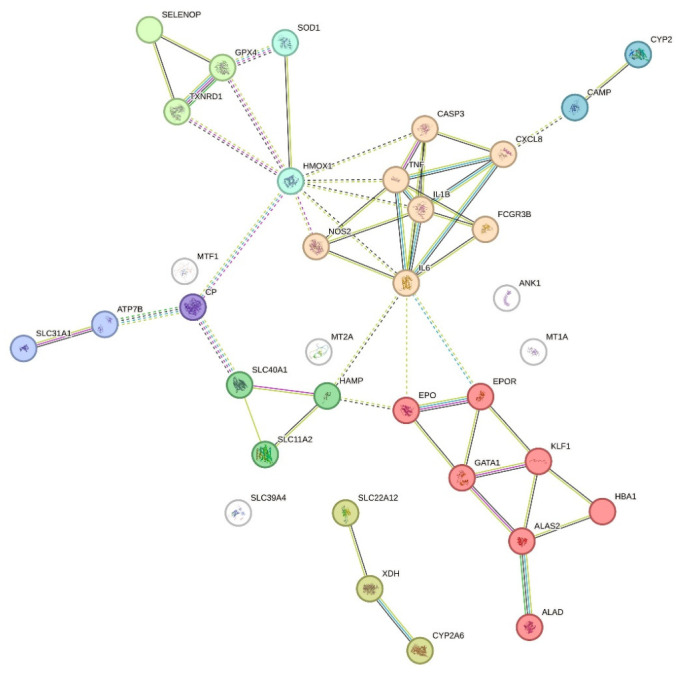
Protein–protein interaction network of the molecular targets of biomarkers examined in the present study, constructed using the STRING database v12.0 (accessed on 23 June, 2026). The complete biomarker-to-protein mapping with biological rationale is provided in Table S3. Node color reflects Markov Cluster Algorithm module assignment. Five functional modules were identified: the inflammatory hub (beige nodes: IL-6, TNF, IL-1β, NOS2, HMOX1, CASP3, CXCL8, FCGR3B), in which IL-6 is the most connected node bridging all other clusters; the erythroid transcription and haem synthesis module (red nodes: GATA1, KLF1, EPO, EPOR, ALAS2, ALAD, HBA1), showing dense experimentally confirmed interactions among erythroid transcription factors and haem biosynthesis enzymes; the copper–iron homeostasis module (green nodes: CP, ATP7B, SLC31A1, SLC40A1, HAMP, SLC11A2), co-clustering ferroportin and hepcidin with ceruloplasmin and copper transport proteins; the selenium antioxidant cascade (light green nodes: SELENOP, GPX4, TXNRD1), with SOD1 (teal) as a bridge node connecting this module to the inflammatory hub; and the uric acid oxidative metabolism module (olive nodes: XDH, SLC22A12, CYP2A6), topologically isolated from the IL-6 hub and connected to the main network by a single edge, consistent with the independent XOR-driven pro-oxidant pathway identified in regression analyses. Vitamin D targets (CYP27B1, CAMP; blue) form a peripheral subgroup. Isolated nodes (gray outline—SLC39A4, MT1A, MT2A, ANK1, MTF1) had no edges meeting the confidence threshold and reflect upstream or structurally adjacent roles (Table S3). Edge color reflects evidence type: black = co-expression; pink = experimentally determined; blue = gene co-occurrence; yellow = text-mining; green = genomic neighborhood. Edge thickness reflects interaction confidence score.

To further contextualize the results of regression models, the molecular targets of all nine biomarkers were mapped onto a STRING protein–protein interaction network (Table S3 and Figure 2).

The network architecture provided convergent support for the regression findings across all nine biomarkers, with the molecular targets of copper and zinc distributed across four of the five modules and both bridge nodes. The inflammatory hub (beige nodes) confirmed IL6 as the master upstream regulator of pathways involving Cu/Zn imbalance, selenium depletion, and hepcidin upregulation simultaneously. The erythroid transcription and haem synthesis module (red nodes) was the most densely connected cluster, with δ-aminolevulinate dehydratase (ALAD) co-clustering with zinc-finger transcription factors rather than with lead-related nodes, explaining both the largest regression effect size for RDW and the non-significance of lead after mutual adjustment. The selenium antioxidant cascade (light green nodes) formed the tightest module immediately downstream of SOD1, confirming that selenium depletion adds to rather than overlaps with Cu/Zn-driven oxidative stress, consistent with independent protective character of selenium. The copper–iron homeostasis module (green, purple, and blue nodes) confirmed that ceruloplasmin ferroxidase failure and hepcidin-driven iron restriction are functionally inseparable and co-regulated by the same IL6 feed-forward loop. Finally, the uric acid module (olive nodes) was an isolated cluster with no direct connection to IL6 or any copper–zinc node. SOD1 and heme oxygenase 1 (HMOX1) (teal bridge nodes) connected the oxidative stress and inflammatory arms. Collectively, this concordance between network topology and regression findings across all nine biomarkers provides convergent evidence from two independent analytical frameworks in support of the oxidative stress network model of Cu/Zn-associated anemia in CKM syndrome.

## 3. Discussion

This cross-sectional analysis of 2391 US older adults with CKM syndrome provides evidence that the serum Cu/Zn ratio is independently associated with increased odds of anemia within the oxidative stress network of CKM syndrome. The biomarkers that correlated with the Cu/Zn ratio, including selenium, cadmium, and RDW, were also those most consistently associated with hemoglobin and prevalent anemia in adjusted regression models, supporting the internal coherence of the redox network hypothesis.

The increased serum Cu/Zn ratio observed in anemic participants, mainly driven by increased copper and decreased zinc concentrations, most likely reflects the molecular footprint of the hyperactivation of IL-6-mediated chronic inflammation, which simultaneously induces hepatic metallothionein, sequestering cytosolic zinc [35], and stimulates the synthesis of ceruloplasmin, raising serum copper levels [36,37]. In CKM patients with deteriorated kidney function, urinary zinc loss and impaired intestinal absorption through Zrt/Irt-like protein 4 (ZIP4) (SLC39A4) may further compound this imbalance [38,39].

The resulting Cu/Zn dysregulation may contribute to anemia through at least three convergent pathways: copper excess may activate MTF1, which upregulates the hepatic transcription of hepcidin, driving functional iron deficiency [40]; simultaneously, elevation of IL-6 induces the activation of metallothionein with subsequent sequestration of cytosolic copper and decreased ATP7B-mediated cuproylation of ceruloplasmin [35,41], thus reducing ferroxidase activity and iron mobilization from enterocytes and reticuloendothelial macrophages [42,43]; finally, concurrent zinc depletion may exacerbate the effects of copper excess, through disruption of the GATA binding protein 1 (GATA1) and Krüppel-like factor 1 (KLF1) zinc-finger domains that govern erythroid transcription and haem synthesis via ALAD, thus reducing the erythroid output [44,45,46,47]. This is confirmed by studies showing that oral zinc supplementation can increase hemoglobin in patients with chronic anemia [48].

Another important finding of the present study is the inverse association between the Cu/Zn ratio and serum selenium. Copper excess suppresses synthesis of selenoproteins GPx1 and GPx4, by interfering with UGA recoding of selenocysteine [49]. Furthermore, chronic inflammation observed in CKM elevates IL-6, a prognostic marker of cardiovascular events [50], which can suppress selenoprotein P, the hepatic protein that distributes selenium to peripheral tissues [29]; additionally, it can induce metallothionein and ceruloplasmin [29], thus leading to selenium deficiency and impairment of copper metabolism. As CKM progresses and kidney function deteriorates, urinary selenium losses and decreased intestinal absorption under uremic enteropathy further deepen this deficit [29].

Selenium deficiency may contribute to anemia, by interfering with an intracellular enzymatic cascade, governed by two sequential antioxidant enzymes, represented by SOD1 and GPx: SOD1 dismutates peroxide to H_2_O_2_, which GPx reduces to water via the glutathione oxidation cycle [23,51]. Selenium is essential for this second step, as selenocysteine incorporation is required for the optimal GPx activity; when selenium is deficient, GPx loses catalytic efficiency and H_2_O_2_ accumulates downstream of SOD1 [29]; the resulting Fenton-mediated generation of hydroxyl radicals drives lipid peroxidation of the erythrocyte membrane [52,53] and may trigger ferroptosis-like membrane collapse [54]. The regression findings are consistent with this pathway: selenium independently protected against both hemoglobin decrease and anemia odds even after full adjustment for the Cu/Zn ratio and all other panel biomarkers.

The positive correlation between the Cu/Zn ratio and RDW may instead reflect the downstream hematological integration of these converging oxidative insults. Anisocytosis, quantified by RDW, generally arises when multiple erythropoietic inputs are simultaneously impaired [33]. In the present context, eryptosis triggered by membrane peroxidation shortens erythrocyte lifespan and drives compensatory release of larger reticulocytes [55,56]. Simultaneously, GATA1/KLF1 disruption produces erythroblasts of variable volume [57,58]; hepcidin-driven iron restriction generates microcytes while reticulocytes remain large, thus widening the size distribution captured by RDW [59]. This explains why RDW carries the largest absolute regression coefficient and the highest odds ratio for anemia of any biomarker in the panel, and why it remains independently significant even after mutual adjustment with the Cu/Zn ratio itself.

The positive correlation between the Cu/Zn ratio and cadmium reflects the known zinc-competing mechanism by which cadmium displaces zinc at metallothionein cysteine residues, further depleting bioavailable zinc [60] and amplifying the Cu/Zn ratio elevation. However, the paradoxical inverse association between cadmium and odds of anemia may be due to nutritional confounding: cadmium accumulates in individuals consuming more shellfish, organ meats, and whole grains, dietary patterns that simultaneously deliver higher selenium, zinc, and protein [61].

Despite showing no significant correlation with Cu/Zn, serum uric acid independently increased anemia odds in our study, demonstrating a parallel oxidative pathway. Under the ischemic and inflammatory conditions of CKM syndrome, xanthine oxidoreductase (XOR) shifts toward its oxidase form [62,63], generating superoxide and H_2_O_2_, alongside uric acid; the resulting ROS may attack erythrocyte membranes through a route independent of the SOD1-GPx cascade [64,65]. Finally, lead, vitamin D, and cotinine showed no independent contributions to anemia after full adjustment, though the positive cotinine–hemoglobin correlation in univariate analysis may be due to smoking-related compensatory polycythemia [66].

Study findings are further corroborated by the protein–protein interaction network constructed using the STRING database, which confirms that the molecular targets of the nine biomarkers converge on five functionally distinct clusters (inflammatory, erythroid, selenium-dependent antioxidant, copper–iron homeostasis, and uric acid-dependent), whose architecture is consistent with the oxidative stress network model linking Cu/Zn dysregulation to anemia in CKM syndrome.

The clinical implications of the present study are grounded in the accessibility of identified biomarkers within this architecture. Serum copper, zinc, and selenium were simultaneously quantified by ICP-DRC-MS from a single standard venipuncture specimen, making the Cu/Zn ratio and selenium concentration immediately accessible in any clinical laboratory with trace metal capabilities; RDW and uric acid were universally available from routine complete blood count and chemistry panels. The convergence of these four independently significant factors on odds of anemia in older adults, through the SOD1–GPx double enzymatic axis, MTF1–hepcidin amplification, GATA-1/KLF1 transcriptional failure, and XOR-derived oxidative stress, identifies multiple pharmacologically accessible intervention points: selenium supplementation to restore GPx activity; zinc supplementation with copper monitoring to correct GATA-1/KLF1 zinc-finger integrity and ALAD haem synthesis capacity without precipitating iatrogenic hypocupremia [40]; and xanthine oxidase inhibition with febuxostat or allopurinol to attenuate the uric acid–mediated oxidative pathway.

Several limitations of this study should be acknowledged. First, the cross-sectional design precluded causal inference and does not allow us to establish the temporal sequence between Cu/Zn imbalance and anemia onset; in this regard, anemia itself could lead to increased Cu/Zn ratio through compensatory erythroid stress and inflammatory responses, which can raise copper levels [67]; second, direct enzymatic activity assays for SOD1 and GPx were not available, so the mechanistic pathway is inferred from biomarker concentrations rather than confirmed at the enzymatic level; third, the subtype of anemia was not differentiated as reticulocyte indices, serum ferritin, and transferrin saturation were not uniformly available across cycles. Fourth, the NHANES metals subsample introduced a selection step that, while probability-based, reduced the effective sample size and may limit generalizability of study findings. Our study has also several strengths: the principal one is the use of a large, nationally representative sample of US adults drawn from three consecutive NHANES cycles; the simultaneous ICP-DRC-MS quantification of copper, zinc, and selenium from a single venipuncture specimen ensures analytical consistency and eliminates inter-assay variability. The availability of a validated CKM staging framework applied to a community-dwelling population [3], combined with a panel of biomarkers spanning antioxidant cofactors, pro-oxidant exposures, RDW, and NLR allows a more comprehensive interrogation of the oxidative stress network than single-biomarker approaches.

## 4. Materials and Methods

### 4.1. Study Design and Population

We performed a cross-sectional analysis of publicly available, de-identified data from the National Health and Nutrition Examination Survey (NHANES), a continuous, nationally representative health and nutrition monitoring program administered by the Centers for Disease Control and Prevention (CDC) through a stratified, multistage, probability cluster-sampling design targeting the non-institutionalized US population.

The NHANES study was conducted in accordance with the Declaration of Helsinki and approved by the National Center for Health Statistics Review Board. Protocol approval was obtained from the National Center for Health Statistics Review Board. This study is a secondary analysis of publicly available NHANES data; therefore, no additional institutional approval was required.

Inclusion criteria for the NHANES study are broad: patients must be non-institutionalized US residents, able to provide written informed consent, and willing to participate in the study. No restrictions were applied by the general NHANES protocol based on disease status, medication use, or laboratory values. For the present analysis, we pooled data from the three consecutive biennial cycles (2011–2012, 2013–2014, and 2015–2016) with available determination of serum copper and zinc. From 7,284 adults with complete data on trace elements across these cycles, we applied sequential restriction criteria to define the analytic sample, including 2391 adults aged 50 years or more, with a diagnosis of CKM stage 1–4, and with available information on anemia prevalence, the other biomarkers, and study covariates. All sampling weights, stratification variables (SDMVSTRA), and primary sampling unit codes (SDMVPSU) were incorporated into every analysis following NCHS analytic guidelines for multi-cycle pooled data; the pooled sample weight for each participant was defined as one-third of the NHANES cycle-specific two-year subsample weight (WTSA2YR) to account for three cycles of equal-duration pooling [68].

### 4.2. Laboratory Measurements

#### 4.2.1. Serum Copper, Zinc, and Selenium

Serum concentrations of copper (µg/dL), zinc (µg/dL), and selenium (µg/L) were quantified simultaneously from a single fasting venipuncture specimen by inductively coupled plasma dynamic reaction cell mass spectrometry (ICP-DRC-MS) at the Division of Laboratory Sciences, National Center for Environmental Health, CDC, Atlanta, GA, using a PerkinElmer ELAN^®^ DRC II mass spectrometer (PerkinElmer, Inc., Norwalk, CT, USA) [69]. Briefly, each serum specimen was diluted in the volumetric ratio 1:1:28 (serum–ultrapure water–aqueous gallium-containing diluent). The analytical principle exploited the coupling of radio-frequency power into a flowing argon stream to generate plasma at 6000–8000 K; the thermal energy vaporized the nebulized liquid sample, atomized all molecules, and ionized the resulting atoms. The ion beam passed sequentially through a focusing lens array, then the dynamic reaction cell (DRC), and finally a quadrupole mass filter to the channel electron multiplier detector. The isotopes monitored were ^64^Zn at m/z 64, ^65^Cu at m/z 65, ^78^Se at m/z 78, and ^71^Ga at m/z 71 (internal standard). The DRC cell was pressurized with ammonia gas (NH_3_) to remove interfering ions. The serum Cu/Zn ratio was calculated as the quotient of serum copper to serum zinc.

The lower limits of detection (LLOD) were 2.5 µg/dL for copper, 2.9 µg/dL for zinc, and 4.5 µg/L for selenium. No participant had values below the LLOD for any of the three analytes [69].

#### 4.2.2. Blood Lead and Cadmium

Whole blood lead (µg/dL) and cadmium (µg/L) were quantified from EDTA-anticoagulated whole blood by ICP-DRC-MS on the PerkinElmer ELAN^®^ DRC II at the CDC National Center for Environmental Health (PerkinElmer, Inc., Norwalk, CT, USA) [70]. Blood samples were diluted in a 1:1:48 ratio (whole blood–ultrapure water–diluent) and measured in standard (vented) mode. LLODs were 0.1 µg/L for cadmium and 0.07 µg/dL for lead. Participants with values below the LLOD were assigned a value of LLOD/√2 in accordance with standard NHANES practice.

#### 4.2.3. Other Laboratory Measures

Serum uric acid (mg/dL), vitamin D (nmol/L), cotinine (ng/mL), hemoglobin (g/dL), RDW (%), and NLR were measured as previously reported [71]. Anemia was defined according to the WHO criteria as hemoglobin <13.0 g/dL in men and <12.0 g/dL in women [69]. Serum creatinine (mg/dL) was included and used to calculate eGFR using the 2021 CKD-EPI creatinine equation without a race variable [70]:

eGFR = 142 × min(Scr/κ, 1)^α × max(Scr/κ, 1)^(−1.200) × 0.9938^Age, where κ = 0.7 (female) or 0.9 (male) and α = −0.241 (female) or −0.302 (male).

The urinary albumin-to-creatinine ratio (ACR = urinary albumin/urinary creatinine × 100, mg/g) was computed from a single voided spot urine specimen. CKM syndrome stages were assigned per the 2023 AHA Presidential Advisory construct using available NHANES variables as surrogates, and classification was previously validated in NHANES and other cohorts [3,4,72]. Details on CKM stage operationalization are reported in Table S4.

### 4.3. Covariates

Demographic variables included age (continuous, years) and sex (male/female). BMI (kg/m^2^) was derived from calibrated stadiometer and scale measurements; obesity was defined as BMI ≥30 kg/m^2^. Comorbidities included cardiovascular disease, defined as congestive heart failure or coronary artery disease, hypertension, type 2 diabetes mellitus, stroke, and cancer; they were ascertained from standardized physician-diagnosis self-report questionnaire items administered by trained interviewers using computer-assisted personal interview, along with laboratory values and medication data.

### 4.4. Statistical Analysis

Continuous variables were reported as mean (SD) and median (IQR) according to their distribution, evaluated through visual inspection and the Shapiro Wilk test; categorical variables were reported as survey-weighted frequencies and percentages. Comparisons of study variables between patients with and without anemia were performed through survey-weighted Student’s *t*-tests for normally distributed continuous variables, the survey-weighted Wilcoxon rank-sum test for skewed continuous variables (cotinine), and Rao–Scott chi-square tests for categorical variables.

Survey-weighted Spearman rank-order correlations between each individual biomarker and either the Cu/Zn ratio or hemoglobin concentration were computed. All *p*-values were two-sided. Benjamini–Hochberg FDR correction was applied to correct for multiple comparisons. A Benjamini–Hochberg adjusted *p*-value < 0.05 was considered statistically significant. Survey-weighted linear regression models were then used to evaluate the association between the Cu/Zn ratio, each oxidative-stress biomarker, and hemoglobin concentration as a continuous outcome. Survey-weighted logistic regression was used to evaluate the association with prevalent anemia as a binary outcome. In both regression types, all nine biomarkers (Cu/Zn ratio, selenium, lead, cadmium, vitamin D, cotinine, uric acid, RDW, and NLR) entered the model as continuous predictors. Covariates in both models were selected a priori based on established clinical risk factors for anemia: Model A included demographic variables (age and sex); Model B additionally included obesity (BMI ≥30 kg/m^2^), chronic diseases (cardiovascular disease, hypertension, diabetes mellitus, cancer, stroke), and advanced CKM (stage III–IV). In the multivariable linear and logistic regression models, FDR correction was applied to the nine biomarker coefficients, while age, sex, and clinical variables were included as adjustment covariates and were not included in FDR correction. FDR-adjusted *p*-values <0.05 were considered statistically significant after correction for multiple comparisons.

To finally contextualize the measured biomarkers within a system biology framework, the molecular targets of all biomarkers were mapped to their principal proteins and enzyme mediators based on curated biochemical interactions (Table S3). A protein–protein interaction network was then built using the STRING database v12.0 [73,74], with a minimum interaction confidence score of 0.700, restricted to experimentally confirmed and literature-curated interaction in *Homo sapiens*. The Markov Cluster Algorithm was applied within STRING to identify functional modules.

All tests were two-sided with a significance threshold of α = 0.05. Statistical analyses were performed in R version 4.3.0 (R Foundation for Statistical Computing, Vienna, Austria) using the survey package (v4.2) for complex-sample design inference.

## 5. Conclusions

In a nationally representative cross-sectional sample of US older adults with CKM stage I-IV, serum Cu/Zn ratio was independently associated with anemia and lower hemoglobin concentration after simultaneous adjustment for a comprehensive panel of redox biomarkers including selenium, uric acid, RDW, lead, cadmium, and cotinine. The biological plausibility of this finding is supported by four non-redundant molecular pathways: impairment of the SOD1–GPx enzymatic cascade; MTF1-driven hepcidin amplification creating a feed-forward loop with the IL-6–ferroportin axis; disruption of GATA-1/KLF1 zinc-finger transcriptional programming governing erythroid maturation and haem synthesis; and XOR-derived oxidative membrane damage operating independently from the SOD1 node. Selenium, RDW, and uric acid were each independently associated with anemia odds, consistent with their mechanistic roles as, respectively, the obligate GPx cofactor downstream of SOD1, the integrative erythrocyte population response to converging oxidative insults, and the terminal marker of the parallel XOR-driven superoxide pathway. The convergence of these biomarkers on anemia risk in CKM identifies pharmacologically actionable targets: selenium repletion to restore GPx activity; zinc supplementation with copper monitoring to correct GATA-1/KLF1 zinc-finger integrity and ALAD haem synthesis capacity without precipitating iatrogenic hypocupremia; and xanthine oxidase inhibition to attenuate the uric acid-mediated oxidative pathway. Prospective trials testing these targeted interventions in CKM patients with elevated Cu/Zn ratio and Erythropoiesis-Stimulating Agent hyporesponsiveness are needed to further confirm these preliminary findings.

## Figures and Tables

**Table 1:** Baseline characteristics of NHANES 2011–2016 participants stratified by anemia status.

Characteristic	All (n = 2391)	No Anemia (n = 2186)	Anemia (n = 205)	*p* Value
Age, years, mean (SD)	63.6 (9.3)	63.2 (9.1)	67.6 (10.2)	<0.001
Female sex, n (%)	1219 (51.0)	1105 (50.5)	114 (55.8)	0.213
Obesity, n (%)	978 (40.9)	895 (40.9)	83 (40.5)	0.986
Cardiovascular disease, n (%)	216 (9.0)	174 (8.0)	42 (20.5)	<0.001
Hypertension, n (%)	1713 (71.6)	1543 (70.6)	170 (82.9)	0.006
Type 2 Diabetes mellitus, n (%)	609 (25.5)	530 (24.2)	79 (38.5)	0.001
Stroke, n (%)	114 (4.7)	92 (4.2)	22 (10.6)	<0.001
Cancer, n (%)	445 (18.6)	400 (18.3)	45 (22.0)	0.329
eGFR, mL/min/1.73 m^2^, mean (SD)	83.0 (18.8)	84.3 (17.5)	69.1 (25.2)	<0.001
ACR ≥ 30 mg/g, n (%)	326 (13.6)	274 (12.5)	52 (25.4)	<0.001
CKM stages III–IV, n (%)	395 (16.5)	318 (14.5)	77 (37.6)	<0.001
Serum copper, µg/dL, mean (SD)	117.5 (24.0)	116.8 (23.2)	126.3 (29.8)	<0.001
Serum zinc, µg/dL, mean (SD)	82.5 (14.7)	83.0 (14.5)	76.5 (15.5)	<0.001
Cu/Zn ratio, mean (SD)	1.5 (0.4)	1.4 (0.4)	1.7 (0.5)	<0.001
Selenium, µg/L, mean (SD)	195.5 (27.7)	197.1 (27.0)	179.0 (29.5)	<0.001
Lead, µg/dL, mean (SD)	1.6 (1.6)	1.6 (1.7)	1.7 (1.3)	0.334
Cadmium, µg/L, mean (SD)	0.5 (0.5)	0.5 (0.5)	0.4 (0.3)	0.013
Vitamin D, nmol/L, mean (SD)	78.9 (31.3)	79.2 (31.3)	75.4 (31.1)	0.174
Cotinine, ng/mL, median (IQR)	0.02 (0.01–0.26)	0.02 (0.01–0.27)	0.02 (0.01–0.19)	0.803
Uric acid, mg/dL, mean (SD)	5.5 (1.4)	5.5 (1.4)	6.0 (1.7)	0.003
NLR, mean (SD)	2.4 (1.3)	2.4 (1.3)	2.5 (1.4)	0.272
RDW, %, mean (SD)	13.5 (1.1)	13.4 (1.0)	14.8 (1.9)	<0.001

ACR = albumin-to-creatinine ratio; CKM = cardiovascular–kidney–metabolic; Cu/Zn = serum copper-to-zinc ratio; eGFR = estimated glomerular filtration rate; RDW = red cell distribution width; NLR = neutrophil-to-lymphocyte ratio.

**Table 2:** Survey-weighted Spearman rank correlations of biomarkers with the serum Cu/Zn ratio and hemoglobin concentration, with Benjamini–Hochberg false discovery correction.

Biomarker	r vs. Cu/Zn	*p* Value	FDR-Adjusted *p* Value	r vs. Hb	*p* Value	FDR-Adjusted *p* Value
Selenium	−0.20	<0.001	<0.001	+0.25	<0.001	<0.001
Lead	−0.02	0.48	0.49	+0.08	<0.001	<0.001
Cadmium	+0.10	<0.001	<0.001	+0.07	<0.001	<0.001
Vitamin D	−0.07	0.01	0.02	−0.02	0.404	0.404
Cotinine	+0.02	0.38	0.49	+0.15	<0.001	<0.001
Uric acid	−0.01	0.66	0.68	+0.09	<0.001	<0.001
RDW	+0.21	<0.001	<0.001	−0.32	<0.001	<0.001
NLR	+0.03	0.17	0.27	+0.04	0.21	0.23

FDR = false discovery rate; Hb = hemoglobin; NLR = neutrophil-to-lymphocyte ratio; RDW = red cell distribution width. FDR-*p* values indicate significance after Benjamini–Hochberg correction (FDR-adjusted *p* < 0.05), applied separately to the 9 simultaneous correlations with Cu/Zn ratio and the 9 correlations with hemoglobin. ‘+’ and ‘−’ symbols indicate the direction of the correlation.

**Table 3:** Survey-weighted linear regression showing the cross-sectional association between Cu/Zn ratio, multiple circulating biomarkers, and hemoglobin concentration, with Benjamini–Hochberg false discovery correction.

Biomarker	Model A, β (95% CI)	FDR-Adjusted *p* Value	Model B, β (95% CI)	FDR-Adjusted *p* Value
Cu/Zn ratio (continuous)	−0.79 (−0.98, −0.60) ***	<0.001	−0.55 (−0.75, −0.35) ***	<0.001
Selenium (per 10 µg/L ↑)	0.08 (0.05, 0.11) ***	<0.001	0.10 (0.07, 0.13) ***	<0.001
Lead	0.009 (−0.16, 0.035)	0.61	0.01 (−0.01, 0.04)	0.44
Cadmium	0.35 (0.24, 0.46) ***	<0.001	0.42 (0.26, 0.58) ***	<0.001
Vitamin D (per 10 nmol/L ↑)	0.002 (−0.015, 0.02)	0.79	0.004 (−0.02, 0.03)	0.68
Cotinine (per 10 ng/mL ↑)	0.006 (0.002, 0.011) **	0.012	0.005 (−0.002, 0.01)	0.19
Uric acid	−0.10 (−0.15, 0.03)	0.67	−0.05 (−0.11, 0.003)	0.09
RDW	−0.31 (−0.37, −0.26) ***	<0.001	−0.31 (−0.39, −0.23) ***	<0.001
NLR	0.05 (−0.005, 0.10)	0.11	0.07 (0.007, 0.13) *	0.06

FDR = False discovery rate; NLR = neutrophil-to-lymphocyte ratio; RDW = red cell distribution width. Model A: age, sex, and all panel biomarkers. Model B: Model A + obesity, cardiovascular disease, hypertension, diabetes, cancer, stroke, CKM stages III–IV, and all panel biomarkers entered simultaneously. * *p* < 0.05; ** *p* < 0.01; *** *p* < 0.001. ↑ indicates a per-interval increment in the concentration of specified biomarkers.

**Table 4:** Survey-weighted logistic regression models showing the association between Cu/Zn ratio, multiple circulating biomarkers and prevalent anemia.

Biomarker	Model A, OR (95% CI)	FDR-Adjusted *p* Value	Model B, OR (95% CI)	FDR-Adjusted *p* Value
Cu/Zn ratio (continuous)	2.88 (1.58–5.27)	<0.001	2.94 (1.61–5.37)	<0.001
Selenium (per 10 µg/L ↑)	0.78 (0.70–0.86)	<0.001	0.76 (0.67–0.86)	<0.001
Lead	1.02 (0.96–1.09)	0.67	1.04 (0.96–1.12)	0.41
Cadmium	0.33 (0.18–0.62)	<0.001	0.23 (0.09–0.56)	<0.001
Vitamin D (per 10 nmol/L ↑)	0.99 (0.92–1.07)	0.85	1.01 (0.92–1.10)	0.94
Cotinine (per 10 ng/mL ↑)	0.99 (0.98–1.02)	0.86	1.00 (0.98–1.02)	0.98
Uric acid	1.23 (1.04–1.45)	0.03	1.33 (1.07–1.65)	0.02
RDW	2.11 (1.71–2.60)	<0.001	2.20 (1.68–2.86)	<0.001
NLR	0.88 (0.72–1.08)	0.33	0.83 (0.67–1.03)	0.14

FDR = false discovery rate; NLR = neutrophil-to-lymphocyte ratio; OR = odds ratio; RDW = red cell distribution width. Model A: age, sex, and all panel biomarkers. Model B: Model A + obesity, cardiovascular disease, hypertension, diabetes, stroke, cancer, CKM stages III-IV, and all panel biomarkers. ↑ indicates a per-interval increment in the concentration of specified biomarkers.

## Data Availability

Publicly available datasets were analyzed in this study. These data can be found at the National Health and Nutrition Examination Survey website: https://wwwn.cdc.gov/nchs/nhanes/ (accessed on 1 June 2026).

## Supplementary Materials

The following supporting information can be downloaded at: https://www.mdpi.com/article/10.3390/ijms27135840/s1.
