# Supplementary material for: Serum Copper-to-Zinc Ratio and Oxidative Stress Are Associated with Anemia in Older Adults with Cardiovascular–Kidney–Metabolic Syndrome
Source: Int J Mol Sci. 2026 Jun 28;27(13):5840. doi: 10.3390/ijms27135840 (PMC13361404; doi:10.3390/ijms27135840)

Table S1. Survey-weighted linear regression showing the cross-sectional association between tertiles of Cu/Zn ratio, multiple circulating biomarkers, and hemoglobin concentration

Biomarker	Model A, β (95% CI)	FDR-adjusted P value	Model B, β (95% CI)	FDR-adjusted P value
Cu/Zn ratio T1	-	-	-	-
T2	-0.22 (-0.36, -0.07)	0.004	-0.29 (-0.49, -0.08)	0.008
T3	-0.30 (-0.48, -0.12)	0.002	-0.27 (-0.48, -00.07)	0.011
Selenium (per 10 μg/L ↑)	0.08 (0.05, 0.11)	<0.001	0.10 (0.07, 0.13)	<0.001
Lead	0.01 (-0.02, 0.03)	0.586	0.01 (-0.02, 0.04)	0.502
Cadmium	0.35 (0.24, 0.46)	<0.001	0.41 (0.25, 0.57)	<0.001
Vitamin D (per 10 nmol/L ↑)	0.01 (-0.01, 0.02)	0.566	0.01 (-0.02, 0.03)	0.511
Cotinine (per 10 ng/mL ↑)	0.01 (0.002, 0.01)	0.007	0.01 (-0.01, 0.01)	0.146
Uric acid	-0.02 (-0.06, 0.02)	0.335	-0.06 (-0.12, -0.01)	0.043
RDW	-0.33 (-0.38, -0.27)	<0.001	-0.33 (-0.41, -0.25)	<0.001
NLR	0.05 (-0.01, 0.10)	0.093	0.07 (0.01, 0.13)	0.043

Notes: FDR = false discovery rate; NLR = neutrophil-to-lymphocyte ratio; RDW = red cell distribution width. Model A: age, female sex, and all panel biomarkers; T = tertile. Model B: Model A + obesity, cardiovascular disease, hypertension, diabetes, stroke, cancer, CKM stages III-IV, and all panel biomarkers. ↑ indicates a per-interval increment in the concentration of specified biomarkers.

Table S2. Survey-weighted logistic regression showing the cross-sectional association between tertiles of Cu/Zn ratio, multiple circulating biomarkers, and hemoglobin concentration

**Biomarker**	**Model A, β (95% CI)**	**FDR-adjusted P value**	**Model B, β (95% CI)**	**FDR-adjusted P value**
Cu/Zn ratio T1	-	-	-	-
T2	2.16 (1.18-3.95)	0.014	5.07 (2.56-9.99)	<0.001
T3	2.07 (1.07-3.99)	0.030	3.39 (1.92-5.99)	<0.001
Selenium (per 10 μg/L ↑)	0.77 (0.70-0.85)	<0.001	0.77 (0.68-0.87)	<0.001
Lead	1.03 (0.97-1.10)	0.339	1.04 (0.95-1.14)	0.372
Cadmium	0.37 (0.21-0.65)	0.001	0.27 (0.12-0.61)	<0.001
Vitamin D (per 10 nmol/L ↑)	0.99 (0.92-1.06)	0.682	1.00 (0.92-1.10)	0.933
Cotinine (per 10 ng/mL ↑)	1.00 (0.98-1.01)	0.665	0.99 (0.97-1.02)	0.577
Uric acid	1.25 (1.06-1.46)	0.008	1.32 (1,07-1.62)	0.011
RDW	2.14 (1.76-2.60)	<0.001	2.24 (1.76-2.85)	<0.001
NLR	0.89 (0.74-1.07)	0.202	0.84 (0.70-1.02)	0.071

Notes: FDR = false discovery rate; NLR = neutrophil-to-lymphocyte ratio; OR = odds ratio; RDW = red cell distribution width; T = tertile. Model A: age, female sex, and all panel biomarkers; Model B: Model A + obesity, heart disease, hypertension, diabetes, stroke, cancer, CKM stages III-IV, and all panel biomarkers. ↑ indicates a per-interval increment in the concentration of specified biomarkers.

Table S3. Mapping of measured biomarkers to their principal protein and enzyme mediators used for STRING protein-protein interaction network construction (Figure 2).

**Measured biomarker**	**Role in study**	**Gene symbols (STRING input)**	**Biological rationale for protein selection**
**Directly measured biomarkers**			
**Serum copper (Cu)**	Primary exposure — Cu/Zn ratio numerator	**SOD1**, **CP**, **ATP7B**, **MT1A**, **SLC31A1**, **MTF1**	SOD1 is the Cu/Zn-dependent cytosolic antioxidant enzyme whose homodimer stability and catalytic cycling require both cofactors. CP is the copper-carrying multicopper ferroxidase essential for $Fe^{2+} \rightarrow Fe^{3+}$ oxidation and transferrin loading. ATP7B mediates hepatic copper export via the trans-Golgi network (cuproylation of apoceruloplasmin). MT1A sequesters cytosolic copper under IL-6 stimulus via metallothionein induction. SLC31A1 (CTR1) is the principal high-affinity copper importer. MTF1 is the copper-sensing metal-regulatory transcription factor driving hepcidin upregulation.
**Serum zinc (Zn)**	Primary exposure — Cu/Zn ratio denominator	**SOD1**, **MT1A**, **SLC39A4**, **GATA1**, **KLF1**, **ALAD**	SOD1 requires zinc for homodimer stability independently of copper. MT1A binds zinc with femtomolar affinity under IL-6 stimulus, sequestering it from enzymatic use. SLC39A4 (ZIP4) is the intestinal zinc importer; its isolation in the STRING network reflects upstream absorption rather than direct erythroid participation. GATA1 and KLF1 are erythroid transcription factors whose C_4-type and C_2H_2-type zinc-finger DNA-binding domains are disrupted by zinc depletion. ALAD (delta-aminolevulinate dehydratase) is the zinc-cofactor-dependent second enzyme of the porphyrin pathway.
**Serum selenium (Se)**	Antioxidant biomarker — independently protective	**GPX1**, **GPX4**, **SELENOP**, **TXNRD1**	GPX1 and GPX4 are selenocysteine-containing glutathione peroxidases that operate enzymatically downstream of SOD1, reducing H_2O_2 and phospholipid hydroperoxides respectively. SELENOP (selenoprotein P) is the hepatic selenium-distribution protein suppressed by IL-6, linking the inflammatory axis to peripheral selenium depletion. TXNRD1 (thioredoxin reductase 1) maintains the thioredoxin redox cycle essential for GPx regeneration. Together these four proteins constitute the SOD1→GPx antioxidant cascade whose co-depletion with the Cu/Zn ratio is demonstrated by the inverse Cu/Zn–selenium Spearman correlation ($r = -0.20$).
**Red cell distribution width (RDW)**	Composite erythrocyte stress index — independently harmful	**ANK1**, **GATA1**, **KLF1**, **EPOR**	ANK1 (ankyrin-1) is the principal erythrocyte membrane scaffold protein linking spectrin to the lipid bilayer; its disruption causes anisocytosis directly. GATA1 and KLF1 regulate the erythroid maturation programme whose failure under zinc depletion and oxidative stress produces erythroblasts of heterogeneous volume. EPOR mediates EPO-driven compensatory reticulocytosis, releasing larger immature cells that widen

Measured biomarker	Role in study	Gene symbols (STRING input)	Biological rationale for protein selection
			RDW. ANK1 appeared as an isolated node in the STRING network at confidence ≥0.700, reflecting its structural rather than enzymatic role.
**Serum uric acid**	Pro-oxidant	**XDH**, **SLC22A12**	XDH (xanthine oxidoreductase) converts under ischaemic/inflammatory CKM conditions from its NAD^+-dependent dehydrogenase form to the O_2-accepting oxidase form, generating superoxide ($O_2{\bullet}^-$) and H_2O_2 while producing uric acid via molybdopterin cofactor-dependent hydroxylation. XDH co-clustered with SLC22A12 and CYP2A6 in a topologically distinct MCL module, separate from the SOD1–IL-6 hub, consistent with an independent parallel pro-oxidant pathway. SLC22A12 (URAT1) is the principal renal urate transporter regulating serum uric acid concentration.
**Serum cadmium (Cd)**	Pro-oxidant	**MT2A**, **HMOX1**, **SLC11A2**, **CASP3**	MT2A (metallothionein 2A) sequesters cadmium at metallothionein-binding sites, competing with zinc and compounding the Cu/Zn imbalance. HMOX1 (heme oxygenase 1) is induced by cadmium-driven oxidative stress and bridges the cadmium arm to the inflammatory hub in the STRING network. SLC11A2 (DMT1) is the divalent metal transporter at which cadmium competes with Fe^{2+} for intestinal and erythroid uptake. CASP3 mediates cadmium-induced apoptosis of erythroid precursors. The inverse association with anemia in adjusted models reflects nutritional confounding: cadmium accumulates in individuals consuming shellfish, organ meats, and whole grains, dietary patterns delivering higher selenium, zinc, and protein.
**Serum lead (Pb)**	Mechanistically connected via haem synthesis	**ALAD**, **ALAS2**, **HBA1**	ALAD is the classical lead-inhibited enzyme of the porphyrin pathway (lead displaces the catalytic zinc at the active site); its presence in the erythroid MCL cluster rather than a separate lead module confirms that zinc depletion, not lead, is the primary driver of ALAD dysfunction in this CKM population. ALAS2 is the erythroid-specific first enzyme of the porphyrin pathway. HBA1 encodes hemoglobin alpha-chain, the terminal product of erythroid haem incorporation. Non-significance in adjusted models reflects confounding by CKM comorbidities and co-adjustment with ALAD-relevant zinc depletion.
**Serum cotinine**	Smoking exposure marker	**CYP2A6**, **NOS2**, **HMOX1**	CYP2A6 is the cytochrome P450 enzyme that converts nicotine to cotinine, marking smoking exposure. NOS2 (inducible nitric oxide synthase) is upregulated by smoking-related oxidative stress, connecting cotinine to the NOS2–IL-6 inflammatory hub in the STRING network. HMOX1 is shared with the cadmium and inflammatory pathways. The positive cotinine–hemoglobin correlation in univariate analysis reflects smoking-

Measured biomarker	Role in study	Gene symbols (STRING input)	Biological rationale for protein selection
			related compensatory polycythemia rather than a protective biological effect; this association was eliminated after full covariate adjustment.
**Serum vitamin D**	Immunomodulatory biomarker	**VDR**, **CAMP**	VDR (vitamin D receptor) suppresses HAMP (hepcidin) transcription through a VDR-response-element in the HAMP promoter and activates EPO gene expression, connecting vitamin D to both the iron-restriction and erythropoietic arms of the network. CAMP (cathelicidin) is the principal VDR target gene, linking vitamin D to innate immunity and the inflammatory module. VDR and CAMP connected to HAMP and EPO in the STRING network, confirming biological plausibility despite non-significance in adjusted models.
**Neutrophil-to-lymphocyte ratio (NLR)**	Composite inflammatory index	**CXCL8**, **IL1B**, **TNF**, **FCGR3B**	CXCL8 (IL-8) drives neutrophil recruitment and is co-regulated with IL-6 in the same inflammatory hub. IL1B (IL-1β) and TNF (TNF-α) are the canonical pro-inflammatory cytokines co-expressed with IL-6, directly bridging the NLR target proteins to the central inflammatory module. FCGR3B (CD16b) is the neutrophil surface Fc receptor quantified in the neutrophil count used to derive NLR. All four proteins co-clustered with IL-6 in the largest MCL module, confirming that NLR captures the same upstream inflammatory signal as IL-6 and hence contributes no independent information after IL-6-mediated confounders are adjusted.
**Other enzymes**			
**Hepcidin**	Iron restriction mediator	**HAMP**	Hepcidin is the master iron-regulatory peptide hormone that induces lysosomal degradation of ferroportin (SLC40A1), blocking iron egress from enterocytes and reticuloendothelial macrophages. In the proposed model, HAMP is simultaneously upregulated by MTF1-driven copper excess and by the IL-6–JAK2–STAT3 inflammatory axis, creating a feed-forward loop. HAMP co-clustered with SLC40A1, CP, and ATP7B in the copper-iron homeostasis MCL module, confirming that iron restriction and copper transport are functionally inseparable at the protein interaction level.
**Ferroportin**	Iron egress transporter	**SLC40A1**	Ferroportin is the sole known cellular iron exporter; its hepcidin-induced degradation is the terminal effector of functional iron deficiency in anemia of inflammation. SLC40A1 connects the copper-ceruloplasmin arm (CP-dependent $Fe^{2+} \rightarrow Fe^{3+}$ oxidation for transferrin loading) to the hepcidin-driven iron restriction arm, making it the convergence point of two independent copper-related anemia mechanisms modelled in this study.

Measured biomarker	Role in study	Gene symbols (STRING input)	Biological rationale for protein selection
**Erythropoietin (EPO)**	Erythropoietic hormone	**EPO**	EPO anchors the erythroid transcription module (GATA1, KLF1, EPOR, ALAS2, ALAD, HBA1) to the canonical erythropoietic response. Its inclusion ensures that the STRING network captures the complete pathway from transcription factor activation (GATA1/KLF1) through haem biosynthesis (ALAD/ALAS2) to hemoglobin production (HBA1), within a biologically coherent erythroid module, without which the module would lack a functional output node.
**Interleukin-6 (IL-6)**	Master upstream inflammatory regulator	**IL6**	IL-6 is the upstream cytokine simultaneously responsible for: (i) metallothionein induction → zinc sequestration → elevated Cu/Zn ratio; (ii) ceruloplasmin synthesis → elevated serum copper; (iii) selenoprotein P suppression → selenium depletion; (iv) STAT3-mediated HAMP upregulation → iron restriction; (v) acute-phase response activating the entire TNF/IL-1β/CXCL8 inflammatory cluster. IL-6 appeared as the most connected node in the STRING network, bridging all five MCL modules, and its inclusion as a mechanistic anchor is essential to explain the topological coherence of the network.

ALAD, delta-aminolevulinate dehydratase; ALAS2, erythroid-specific aminolevulinate synthase 2; ANK1, ankyrin-1; ATP7B, copper-transporting ATPase 2; CAMP, cathelicidin antimicrobial peptide; CASP3, caspase-3; CP, ceruloplasmin; CXCL8, C-X-C motif chemokine ligand 8 (IL-8); CYP2A6, cytochrome P450 family 2 subfamily A member 6; EPO, erythropoietin; EPOR, erythropoietin receptor; FCGR3B, Fc gamma receptor IIIb (CD16b); GATA1, GATA binding protein 1; GPX1/4, glutathione peroxidase 1/4; HAMP, hepcidin antimicrobial peptide; HBA1, hemoglobin subunit alpha 1; HMOX1, heme oxygenase 1; IL1B, interleukin 1 beta; IL6, interleukin 6; KLF1, Krüppel-like factor 1 (erythroid); MCL, Markov Cluster Algorithm; MT1A/MT2A, metallothionein 1A/2A; MTF1, metal regulatory transcription factor 1; NLR, neutrophil-to-lymphocyte ratio; NOS2, nitric oxide synthase 2; RDW, red cell distribution width; SELENOP, selenoprotein P; SLC11A2, solute carrier family 11 member 2 (DMT1); SLC22A12, solute carrier family 22 member 12 (URAT1); SLC31A1, solute carrier family 31 member 1 (CTR1); SLC39A4, solute carrier family 39 member 4 (ZIP4); SLC40A1, solute carrier family 40 member 1 (ferroportin); SOD1, superoxide dismutase 1; STRING, Search Tool for the Retrieval of Interacting Genes/Proteins; TNF, tumor necrosis factor; TXNRD1, thioredoxin reductase 1; VDR, vitamin D receptor; XDH, xanthine dehydrogenase.

Table S4. NHANES Variable Mapping for CKM Syndrome Staging.

CKM Stage	Criterion	Cutoff / Definition
Stage 0	Absence of overweight/obesity and metabolic risk	BMI <25 kg/m² (non-Asian) or <23 kg/m² (Asian)<br>Waist <88/<102 cm (women/men, non-Asian) or <80/<90 cm (women/men, Asian)<br>No criteria met for Stages 1–4
Stage 1	Overweight/obesity OR prediabetes	BMI ≥25 kg/m² (non-Asian) or ≥23 kg/m² (Asian), OR<br>Waist ≥88/≥102 cm (women/men, non-Asian) or ≥80/≥90 cm (women/men, Asian), OR<br>HbA1c 5.7–<6.5% OR fasting glucose 100–<126 mg/dL
Stage 2	Metabolic risk factors OR moderate-to-high-risk CKD (KDIGO)	Hypertension: self-report OR SBP ≥130 / DBP ≥80 mmHg OR BP-lowering medication<br>Diabetes: self-report OR HbA1c ≥6.5% OR fasting glucose ≥126 mg/dL OR insulin/antidiabetic medication<br>Fasting serum triglycerides ≥135 mg/dL<br>Metabolic syndrome: ≥3 of (elevated waist, HDL <40/<50 mg/dL men/women, fasting serum triglycerides ≥150 mg/dL, elevated BP, prediabetes)<br>Moderate-to-high-risk CKD: eGFR 45–59 + ACR ≥30 mg/g, OR eGFR <45 (any ACR)
Stage 3	Very high-risk CKD (KDIGO) OR high predicted 10-year CVD risk	Very-high-risk KDIGO CKD: eGFR <30 (any ACR), OR eGFR 30–59 + ACR ≥300 mg/g, OR eGFR <60 + ACR ≥300 mg/g<br>OR 10-year CVD risk ≥20% by AHA PREVENT equations (adults 30–79 yrs; those ≥80 yrs assigned age=79)
Stage 4	Established (overt) cardiovascular disease	Self-reported diagnosis of any of: coronary heart disease, angina, heart attack, heart failure, or stroke

ACR = albumin-to-creatinine ratio; BMI = body mass index; BP = blood pressure; CKD = chronic kidney disease; CKM = cardiovascular-kidney-metabolic; CVD = cardiovascular disease; DBP = diastolic blood pressure; eGFR = estimated glomerular filtration rate (calculated using the race-free CKD-EPI 2021 creatinine equation); HDL = high-density lipoprotein; KDIGO = Kidney Disease Improving Global Outcomes; NHANES = National Health and Nutrition Examination Survey; PREVENT = Predicting Risk of CVD EVENTs; SBP = systolic blood pressure; TG = triglycerides. Row shading reflects CKM syndrome severity.

Reference: Aggarwal R, Ostrominski JW, Vaduganathan M. Prevalence of cardiovascular-kidney-metabolic syndrome stages in US adults, 2011–2020. JAMA. 2024. doi:10.1001/jama.2024.6892

Figure S1 Survey-weighted Spearman rank correlations between circulating antioxidant and pro-oxidant biomarkers and the serum Cu/Zn ratio (blue bars) and hemoglobin concentration (red bars). Horizontal bars represent survey-weighted Spearman correlation coefficients (r) between each circulating biomarker and the serum Cu/Zn ratio (left panel, red) or hemoglobin concentration (right panel, blue). Bars extending to the left indicate inverse correlations; bars extending to the right indicate positive correlations. Asterisks denote statistical significance after Benjamini–Hochberg false discovery rate correction: *** FDR-adjusted $p < 0.001$. Biomarkers not reaching statistical significance after FDR correction are shown without asterisks.

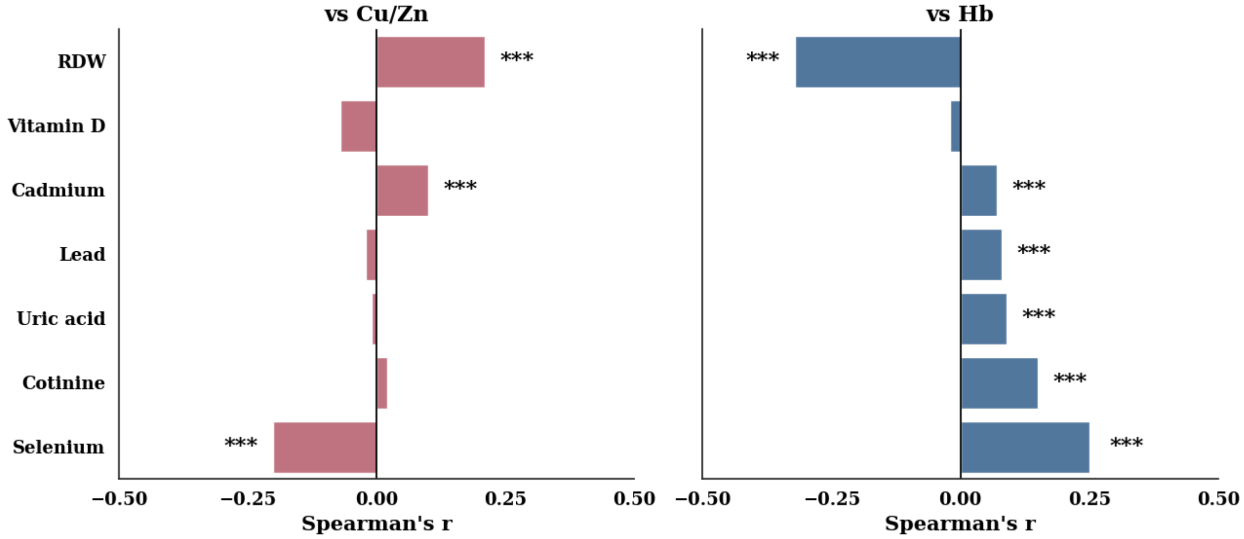

Supplement: Supplementary file 1 [file ijms-27-05840-s001.zip › ijms-4387500-supplementary.pdf]
